# Bioinformatics profiling of NECTIN4 in lung cancer and comparative evaluation of NECTIN4-targeted ⁶⁸Ga-N188 and ¹⁸F-FDG PET/CT

**DOI:** 10.1186/s12967-026-08152-8

**Published:** 2026-04-27

**Authors:** Yuqi Wang, Xin Zhou, Jinchuan Chen, Futao Liu, Yutao Li, Yuan Li, Kezhong Chen, Jun Wang, Xing Yang, Nan Li

**Affiliations:** 1 Department of Nuclear Medicine, Peking University People’s Hospital, No. 11 Xizhimen South Street, Xicheng District, Beijing, 100044 China; 2 Key laboratory of Carcinogenesis and Translational Research (Ministry of Education), Beijing Key Laboratory of Research, Investigation and Evaluation of Radiopharmaceuticals, NMPA Key Laboratory for Research and Evaluation of Radiopharmaceuticals (National Medical Products Administration), Department of Nuclear Medicine, Peking University Cancer Hospital & Institute, No. 52 Fucheng Road, Haidian District, Beijing, 100142 China; 3 Department of Thoracic Surgery, Thoracic Oncology Institute, Peking University People’s Hospital, No. 11 Xizhimen South Street, Xicheng District, Beijing, 100044 China; 4 Research Unit of Intelligence Diagnosis and Treatment in Early Non-small Cell Lung Cancer, Chinese Academy of Medical Sciences, 2021RU002, Peking University People’s Hospital, Beijing, 100044 China

**Keywords:** NECTIN4, Bioinformatics, ^68^Ga-N188, PET/CT, Genome instability, Lung cancer

## Abstract

**Background:**

The nectin cell adhesion molecule 4 (NECTIN4) has been implicated in tumor progression and immune evasion, yet its role and translational targeted imaging potential in lung cancer remain unclear. Therefore, this study aims to elucidate the significance of NECTIN4 by integrating multi-omics analyses, and to evaluate the diagnostic efficacy of the NECTIN4-targeted PET/CT imaging in lung cancer.

**Methods:**

Transcriptomic and proteomic datasets from The Cancer Genome Atlas (TCGA), Genotype-Tissue Expression (GTEx), Gene Expression Omnibus (GEO) and other bioinformatic tools were used to characterize NECTIN4 expression, genomic alterations, epigenetic regulation, and prognostic relevance in lung cancer. Subsequently, in a prospective clinical cohort study involving 20 patients with suspected primary lung cancer, paired PET/CT imaging using ^68^Ga-N188 and ^18^F-FDG was conducted. Diagnostic performances were assessed by quantitatively comparing the tumor-to-blood pool ratio between malignant and inflammatory lesions.

**Results:**

Bioinformatics analyses indicated that NECTIN4 was significantly upregulated across multiple cancer types and correlated with genomic instability and poor prognosis in non-small cell lung cancer (NSCLC). NECTIN4 expression was positively associated with DNA methyltransferases and RNA modifications, suggesting that it may be regulated by epigenetic and post-transcriptional. As for NECTIN4-targeted imaging, ⁶⁸Ga-N188 PET/CT exhibited superior specificity (100% vs. 50%) and comparable sensitivity (87.5% vs. 93.8%) to ¹⁸F-FDG PET/CT in differentiating malignant from inflammatory lung lesions, but with lower sensitivity (42.2% vs. 100.0%) for detecting lymph node metastases and fewer identified distant metastatic lesions (21 vs. 51).

**Conclusion:**

Integrated bioinformatics analyses prove that overexpression of NECTIN4 is associated with occurrence and progression of lung cancer. Further preliminary clinical translation study suggests the potential of NECTIN4-targeted radiotracer ⁶⁸Ga-N188 to aid in the differential diagnosis of lung cancer, highlighting a promising clinical application warrants further validation.

**Supplementary Information:**

The online version contains supplementary material available at 10.1186/s12967-026-08152-8.

## Background

Lung cancer—the most prevalent malignancy globally—remains the leading cause of cancer-related mortality, leading to about 18.4% of all cancer deaths. Despite advances in therapeutic strategies, the 5-year overall survival rate remains 10% to 15%, primarily due to late-stage diagnosis and the complexity of therapeutic management [1, 2]. As of now, ¹⁸F-FDG PET/CT is a fundamental imaging instrument in the clinical assessment of lung cancer, and is extensively utilized in the diagnosis, staging, and assessment of treatment outcomes in non-small cell lung cancer (NSCLC) and small cell lung cancer (SCLC) [3, 4]. However, ^18^F-FDG PET/CT exhibits limited specificity to differentiate between malignant and inflammatory lesions, and this poses significant challenges in precision medicine. This limitation demonstrates the necessity to design novel PET tracers with higher specificity that will provide more information about tumor biological characteristics to enable a personalized treatment approach and enhance patient outcomes in lung cancer.

Nectin cell adhesion molecule 4 (NECTIN4) is an immunoglobulin-like cell adhesion transmembrane protein, a subgroup of nectin, a type I protein family of cell adhesion molecules. Under normal circumstances, NECTIN4 is strongly expressed in the placenta and embryo, with minimal expression observed in healthy adult tissues and organs. It controls cellular behavior by participating in signaling pathways like the PI3K-Akt signaling pathway and is essential in keeping cell-cell adhesions and tight junctions [5–7]. Many studies have revealed in recent years that NECTIN4 is overexpressed in about two-thirds of patients with NSCLC. And cell lines of SCLC are generally less express NECTIN4 than NSCLC cell lines [8]. Furthermore, NECTIN4 was found to be strongly associated with clinicopathological characteristics, including the size of the tumor, the disease stage, and the existence of distant metastases, and is an independent prognostic variable of overall survival (OS) in NSCLC [9–11]. However, the expression patterns, molecular and cellular interactions and regulatory mechanisms of NECTIN4 in lung cancer still poorly understood. Thus, a comprehensive bioinformatics analysis of NECTIN4 expression in lung cancer could provide further insights into its role in tumorigenesis and its potential as a diagnostic biomarker for lung cancer.

With the approval of Enfortumab Vedotin (EV)—the first antibody-drug conjugate (ADC) targeting NECTIN4—by the U.S. Food and Drug Administration (FDA) and the European Medicines Agency for the treatment of urothelial carcinoma (UC), the clinical significance of NECTIN4 has gained increasing attention. The indication of EV is currently on the rise as several clinical trials are currently underway. At the same time, there are increasingly diverse drugs against NECTIN4 which are being developed and gradually emerging [12–16].

With regards to non-invasive, dynamic, whole-body NECTIN4 expression measurements, a number of NECTIN4 imaging probes, such as monoclonal antibody-derived ones, have been created and published [17–19]. However, clinical translation of these probes is still challenged because of constraints like slow systemic clearance and limited tumor penetration in solid malignancies. Among such probes, ^68^Ga-N188, a NECTIN4-targeted radiotracer based on a bicyclic peptide, exhibits great sensitivity and specificity in detecting NECTIN4-expressing lesions in patients with advanced UC [20]. A head-to-head comparison with ^18^F-FDG PET/CT revealed that ^68^Ga-N188 not only effectively detects lesions but also quantitatively assesses membranous NECTIN4 expression levels across various solid tumors, providing preliminary evidence of its clinical utility [21]. Nevertheless, that study included only two patients with NSCLC, leaving the potential of NECTIN4-targeted PET imaging in lung cancer largely unexplored.

This study aims to conduct an integrative study combining multi-omics bioinformatics profiling validation to investigate NECTIN4 expression patterns, genomic alterations, epigenetic regulation, and their associations with clinical outcomes in lung cancer. Additionally, a head-to-head comparison of NECTIN4-targeted ⁶⁸Ga-N188 and ¹⁸F-FDG PET/CT was performed in patients with suspected primary lung cancer, aiming to evaluate the performance of NECTIN4 as a diagnostic imaging biomarker for lung cancer.

## Methods

### Bioinformatic data collection and processing

Bioinformatic analyses in this study were conducted using publicly available, curated databases. Differential expression of the NECTIN4 gene across various cancer types was analyzed using the TIMER database (http://timer.cistrome.org/). RNA sequencing data and corresponding clinical data were obtained from The Cancer Genome Atlas (TCGA) and Genotype-Tissue Expression (GTEx) databases via UCSC Xena (https://xenabrowser.net/datapages/). Cancer proteomics data were retrieved from the Clinical Proteomic Tumor Analysis Consortium (CPTAC) database. The Lung Cancer Explorer (LCE) database (https://lce.biohpc.swmed.edu/) was utilized to conduct a meta-analysis of OS hazard ratio in patients with lung cancer. Furthermore, single-cell RNA-sequencing (scRNA-seq) datasets for lung cancer (E-MTAB-6149, GSE127465, GSE162498, and GSE210347) were retrieved from the Gene Expression Omnibus (GEO) database. Relevant datasets were selected based on their relevance to lung cancer and NECTIN4-associated molecular mechanisms. Differences in NECTIN4 mRNA expression across disease states (tumor vs. normal) and individual cancer stages were analyzed using the R package *ggplot2* and visualized with box plots and paired-sample wiring diagrams.

### Genomic alteration and mutational burden analyses

Pan-cancer analyses of genomic mutation, amplification, and deep deletion frequencies were performed using the Cancer Type Summary module of cBioPortal [22]. Tumor mutational burden (TMB) and mutant-allele tumor heterogeneity (MATH) were assessed using the R package *maftools*, while ploidy and microsatellite instability (MSI)-related data were obtained from previous reports [23]. Correlations between these genomic characteristics and NECTIN4 expression were subsequently analyzed.

### DNA methylation analyses and epigenetic modification analyses

Data derived from cBioPortal was used to access the correlations between the methylation of NECTIN4 and pan-cancer. Expression data for the NECTIN4 gene and 44 tri-class RNA modifications N1-methyladenosine (m^1^A), 5-methylcytosine (m^5^C), and N6-methyladenosine (m^6^A) modifying genes from the UCSC. The Pearson correlation between NECTIN4 and the marker genes were assessed with the R package.

### ^68^Ga-N188 production

The NECTIN4-targeting ligand N188 was purchased from Shanghai Apeptide Co., Ltd (Shanghai, China) and purified using high-performance liquid chromatography (HPLC) to > 95% purity (Supplementary Fig. 1–2). Radiosynthesis of ^68^Ga-N188 was achieved in a one-step reaction within 15 min based on a previous established protocol [20]. Radiochemical purity was evaluated using radio-HPLC and confirmed at > 95% purity (Supplementary Fig. 3).

### Western blot analysis

Total protein was extracted from tissue using prechilled radioimmunoprecipitation assay buffer containing a protease inhibitor cocktail and measured using bicinchoninic acid assay. Equal amounts of protein samples were separated on a 7.5% sodium dodecyl sulfate–polyacrylamide gel electrophoresis gel and transferred onto 0.45 μm polyvinylidene fluoride membranes. Membranes were blocked with 5% non-fat milk for 1 h, then incubated overnight at 4℃ with primary antibodies, including anti-NECTIN4 (1:2000; Proteintech, Cat. No. 21903-1-AP) and anti-rabbit β-actin (1:100000; Proteintech, Cat. No. 20536-1-AP). Immunoblots were visualized and analyzed using an automated chemiluminescence imaging system (5200 Multi, Tanon, China). The band of β-actin was used as an internal standard to normalize the results and analyzed using Image J software.

### Immunohistochemical analysis

Paraffin-embedded lung tumor and matched adjacent non-tumorous tissues were obtained from patients of department of thoracic surgery, in accordance with institutional ethical guidelines. Tissue sections were fixed in 4% paraformaldehyde, and 4 μm thick slices were prepared from the formalin-fixed, paraffin-embedded blocks and subsequently dewaxed. Using an antigen retrieval solution, sections were pretreated in a microwave oven at medium power for 8 min until boiling, then at medium-low power for 7 min. They were then blocked with 0.3% hydrogen peroxide and goat serum, rinsed with Tris-buffered saline, and incubated with anti-Nectin4 antibody (1:1000) at 4 °C overnight. The sections were subsequently incubated with a horseradish peroxidase-conjugated secondary antibody at room temperature, stained with 3,3’-diaminobenzidine, and counterstained with hematoxylin for 3 min. Images were captured using an optical microscope and analyzed using Image J software.

### Patient information

The Medical Ethics Committee of Peking University Cancer Hospital approved this prospective study (2022KT37-ZY01). Written informed consent was obtained from all participants. The trial was registered at www.clinicaltrialsregister.eu (Trial Identifier: NCT06648317).

Patients with lung lesions were consecutively recruited between September 2024 and December 2024. Inclusion criteria were: (1) presence of lung lesions; (2) normal kidney, liver, and bone marrow hemopoietic function; and (3) an ECOG performance status of 0–1. Exclusion criteria included: (1) prior chemotherapy or radiotherapy; (2) refusal to undergo paired baseline ^68^Ga-N188 PET/CT and ^18^F-FDG PET/CT within 1 week. Clinical and pathological characteristics of the patients were recorded.

### Position emission tomography/computed tomography acquisition

Patients received an intravenous injection of ^68^Ga-N188 (1.9–3.7 MBq/kg) and were instructed to drink 800–1500 mL of water. PET/CT was conducted 1 h post-injection using a 194-cm-long axial field of view (FOV) total-body PET/CT (uEXPLORER, United Imaging Healthcare, Shanghai, China). Acquisition time was 5 min. Image reconstruction was conducted using the ordered subset expectation maximization algorithm with two iterations, 20 subsets, a 192 × 192 matrix, and a 600 mm FOV. The slice thickness was 2.886 mm. Attenuation and scattering corrections were applied, along with point spread function and time-of-flight reconstruction. No post-filtering was applied. The attenuation corrected CT was acquired using 120 kV with a modulated current of approximately 75 mA. Vital signs were recorded before injection, throughout the screening period, and 2 h after the PET/CT scan.

Patients were required to fast for at least 6 h before the ^18^F-FDG PET/CT scans to maintain normal blood glucose levels (4.4–9.3 mmol/L). The intravenous dose was body weight-based (3.7 MBq/kg), followed by a 1 h rest before imaging. Acquisition conditions were consistent with those used for the ^68^Ga-N188 PET/CT.

### PET/CT image analysis

Post-processing of images was conducted using a vendor-provided software (Multi-Modality Workplace, United Imaging, China). Two nuclear medicine physicians with 5–10 years of diagnostic experience, blinded to prior imaging and pathological findings, independently reviewed all images. Discrepancies were resolved by a third physician with 15–20 years of diagnostic experience.

The volume of interest for the primary pulmonary tumor on ^18^F-FDG PET/CT and ^68^Ga-N188 PET/CT images was manually delineated to include the entire target lesion while excluding surrounding tissues and organs. The maximum standardized uptake value (SUV_max_) of the lesions and the mean SUV (SUV_mean_) of the blood pool—derived from uptake in the descending aorta (used as background) were recorded. The tumor-to- blood-pool ratio (TBR) was calculated as tumor SUV_max_/BP SUV_mean_. Metastatic lesions were confirmed via pathology or follow-up imaging. A lesion was considered positive for metastasis if its uptake exceeded the physical uptake of surrounding tissues.

### Statistical analysis

R software (version 4.2.1) was utilized for statistical analyses of the bioinformatics data. Group comparisons were conducted using one-way analysis of variance (ANOVA) or Student’s t-test, as appropriate. Survival analyses were conducted using Kaplan–Meier curves with log-rank tests or Cox proportional hazards regression models. Correlations between variables were assessed using Pearson or Spearman correlation coefficients.

Statistical analyses of the data of the patients were conducted using IBM SPSS Statistics (version 25.0) and GraphPad Prism (version 8.0). Measurement data were expressed as mean ± standard deviation, while categorical variables were presented as frequencies and percentages. Differences in SUV_max_ values among lesions with different pathological types on ^68^Ga-N188 PET/CT were analyzed using one-way ANOVA. The normality of continuous variables was assessed using the Shapiro–Wilk test. For non-normally distributed data, the Mann–Whitney U test was utilized. Data distributions were visualized using histograms. The predictive value of the uptake parameters for differentiating malignant and benign lesions was analyzed using the area under the receiver operating characteristic (ROC) curve, with the cut-off value determined based on the Youden index. The diagnostic performance metrics were calculated. For differential diagnosis of primary lung lesions, the diagnostic metrics were calculated along with their 95% confidence intervals using the Clopper-Pearson exact method. A *p-value of* < 0.05 was considered statistically significant.

## Results

### Expression of NECTIN4 in multiple cancers and lung cancer

**Fig. 1:**
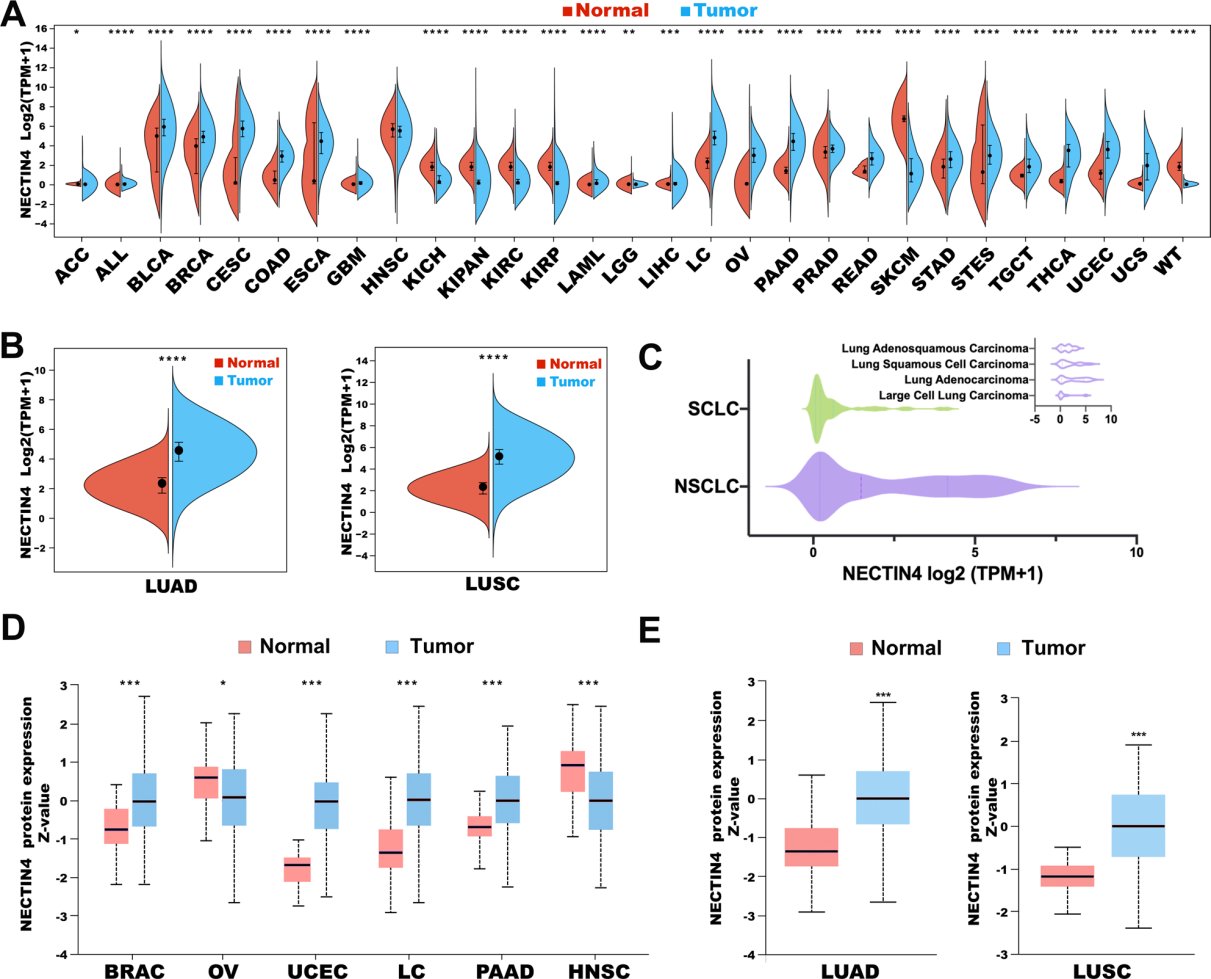
Variation in NECTIN4 expression between tumor and normal samples. (**A**, **B**) Comparison of NECTIN4 mRNA expression in tumors versus normal tissues using TCGA and GTEx datasets. (**C**) Expression profiles across lung cancer cell lines, showing elevated levels in NSCLC than in SCLC. (**D**, **E**) NECTIN4 protein levels in primary tumors and normal tissues, analyzed using the UALCAN database. *P < 0.05, **P < 0.01, and ***P < 0.001

Using RNA sequencing data from the TCGA and GTEx databases for systematic analysis, we investigated the expression of NECTIN4 mRNA across multiple cancer types. Differential expression analysis revealed significantly dysregulation of NECTIN4 mRNA in 16 tumor types, including lung cancer, compared with adjacent normal tissues (Fig. 1A and B). NECTIN4 was significantly overexpressed in lung squamous cell carcinoma (LUSC) and lung adenocarcinoma (LUAD) in the TCGA dataset. Expression levels increased progressively across disease stages (Supplementary Fig. 4).

Consistent with the above findings, NECTIN4 protein was overexpressed in lung cancer, breast invasive carcinoma (BRCA), uterine corpus endometrial carcinoma (UCEC), pancreatic adenocarcinoma (PAAD), with particularly significant in NSCLC (LUAD and LUSC; Fig. 1D and E). Further analysis of 196 lung cancer cell lines also revealed that NECTIN4 expression was markedly elevated in two primary clusters corresponding to NSCLC and SCLC, particularly within the NSCLC group (Fig. 1C).

### Single-cell landscape of NECTIN4 expression in lung cancer

**Fig. 2:**
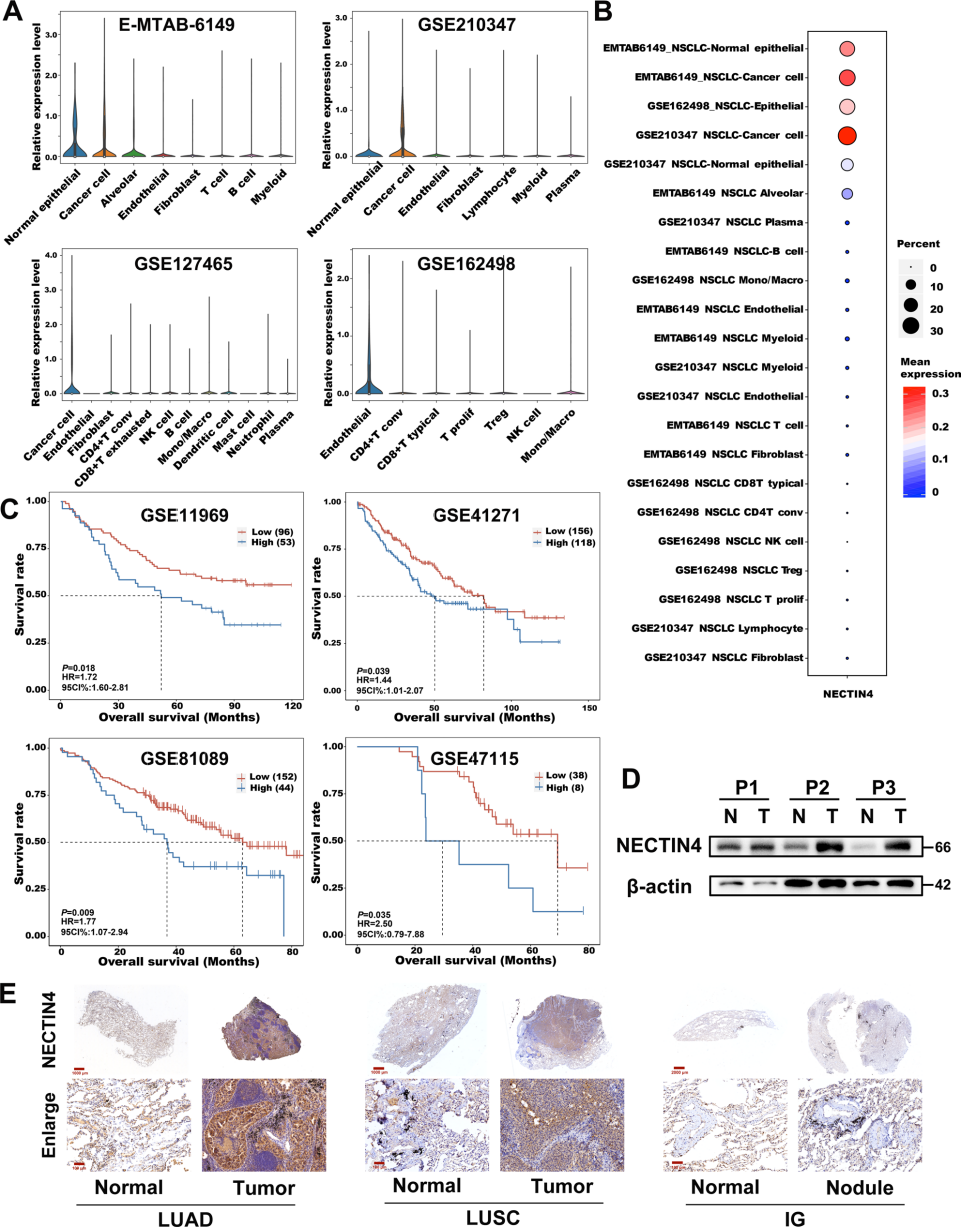
Prognostic relevance and cellular distribution of NECTIN4 in lung cancer. (**A**, **B**) Expression pattern of NECTIN4 in human NSCLC scRNA-seq datasets. (**C**) Western blot image of the tumor tissues in patients with lung cancer. (**D**) IHC staining of the tumor slices in patients with lung cancer. (**E**) Relationship between NECTIN4 expression and OS in patients with NSCLC

By analyzing scRNA-seq transcriptomic data obtained from lung tumor specimens and adjacent non-tumor tissues across multiple datasets (E-TMAB-6169, GSE127465, GSE162498, and GSE210347), we further elucidated the role of NECTIN4 in lung cancer (Fig. 2A and B). The cells were categorized into distinct clusters, including cancer cells, epithelial cells, alveolar cells, and immune cells based on established cell-type markers. NECTIN4 was detected in all clusters, showing highest expression levels observed in cancer cells and lower expression levels in lymphocytes and fibroblasts.

### Molecular validation of NECTIN4 overexpression and correlation with poorer prognosis

NECTIN4 protein expression in NSCLC was evaluated using immunohistochemistry (IHC) and western blot analysis. The results showed that NECTIN4 protein was localized in membranous, and its elevated expression was observed in tumor sections compared to inflammatory and normal lung tissues (Fig. 2D). The findings of the western blot analysis were in agreement with the findings described above (Fig. 2C, Supplementary Fig. 5).

Survival analyses were performed using datasets GSE11969, GSE41271, GSE81089, and GSE47115. It showed that increased NECTIN4 expression had a negative relationship with the OS of patients with NSCLC (Fig. 2E). This association was also confirmed by a meta-analysis of the LCE database, which revealed a stable correlation between high NECTIN4 expression and poor outcomes in NSCLC (Supplementary Fig. 6).

### Genomic alterations and genomic instability in NECTIN4 expression

**Fig. 3:**
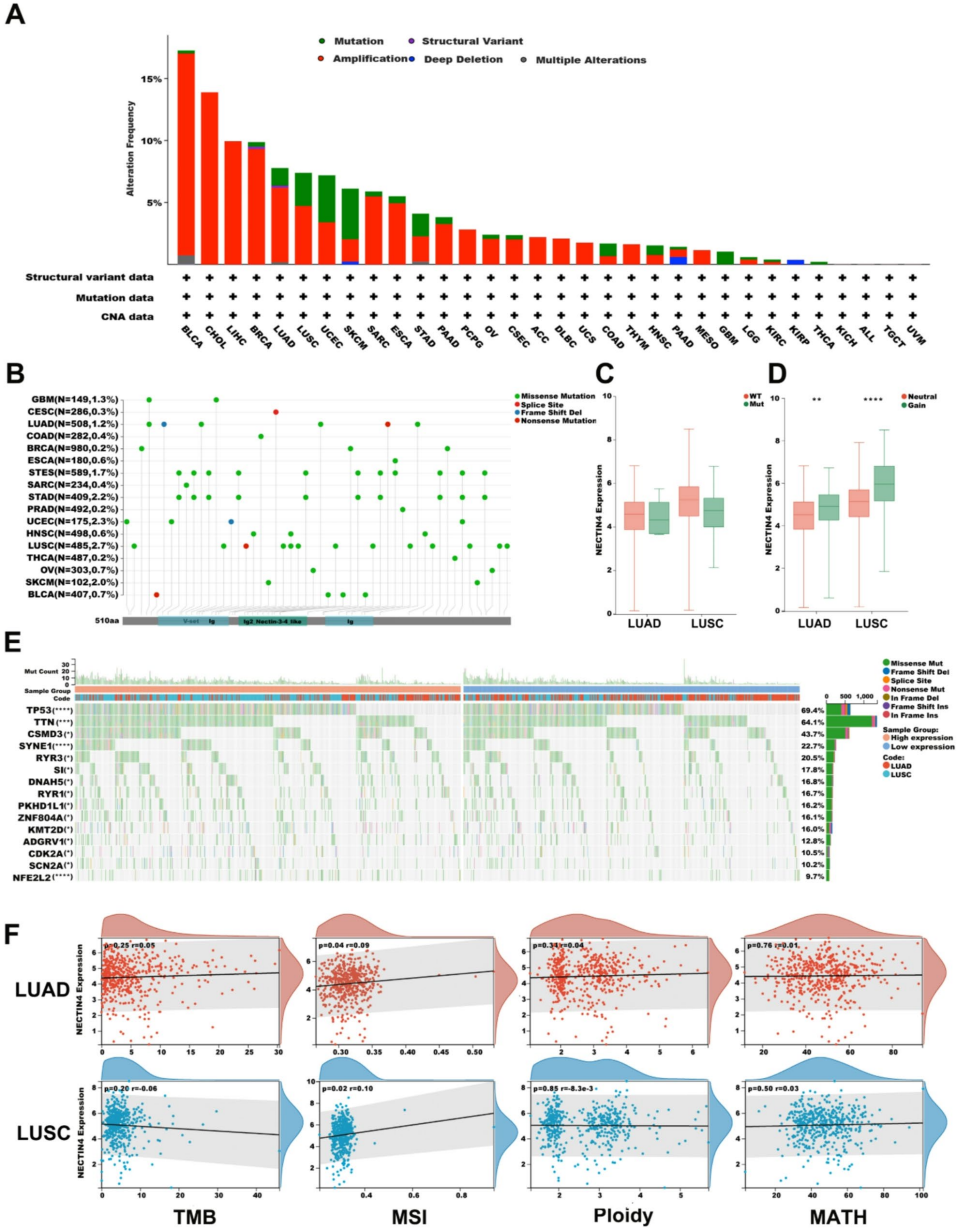
Correlation between genomic alterations and NECTIN4 expression. (**A**) Different alteration types of NECTIN4 alterations of pan-cancer in TCGA datasets, including mutation, structural variant, amplification, deep deletion, and multiple alterations. (**B**) The pan-cancer NECTIN4 mutational landscape, including missense, frameshift deletion, and splice site mutations. (**C**, **D**) Comparison of NECTIN4 mRNA expression levels according to SNVs and CNVs in LUAD and LUSC. (**E**) Mutation co-occurrence patterns associated with NECTIN4 in LUAD and LUSC. (**F**) Correlation analyses of NECTIN4 expression with TMB, MSI, ploidy, and MATH

To assess potential genome-level alterations of NECTIN4 in cancers, we conducted a pan-cancer analysis of NECTIN4 copy number variations (CNVs) and single nucleotide variants (SNVs). The genomic mutation landscape showed that NECTIN4 exhibits frequent genetic alterations across various types of tumors and, most notably, in NSCLC (Fig. 3A, B). NECTIN4 amplification was most frequently detected in multiple cancers, followed by mutation and deep deletion. It is also interesting to note that the frequency of NECTIN4 alteration in LUSC and LUAD is above 10%. Additionally, NECTIN4-gained samples were characterized by elevated NECTIN4 mRNA, indicating that copy number variations might be used to regulate its expression (*P* < 0.0001) (Fig. 3C-D).

Further to simplify the genomic background of NECTIN4 expression, we analyzed patterns of co-occurring mutations in LUAD and LUSC (Fig. 3E). Results revealed that high expression of NECTIN4 was frequently co-expressed with mutations in established cancer-associated genes including TP53, TTN, RYR1, NFE2L2. The NECTIN4 high-expression and low-expression groups exhibited significant differences in mutation profiles and transcriptomic characteristics, showing a different pattern of clustering. On the other hand, correlations between NECTIN4 and TMB, MSI, MATH and ploidy were also evaluated given the abundance of such mutations in pan-cancer (Fig. 3F, Supplementary Fig. 7). NECTIN4 was positively correlated with MSI, and this was significant in both LUAD and LUSC. Correlation analysis further indicated that NECTIN4 expression was positively correlated with TMB, ploidy, and MATH, albeit with modest correlation coefficients.

### Analyses of NECTIN4 with DNA methylation and cancer cell stem-like characteristics

**Fig. 4:**
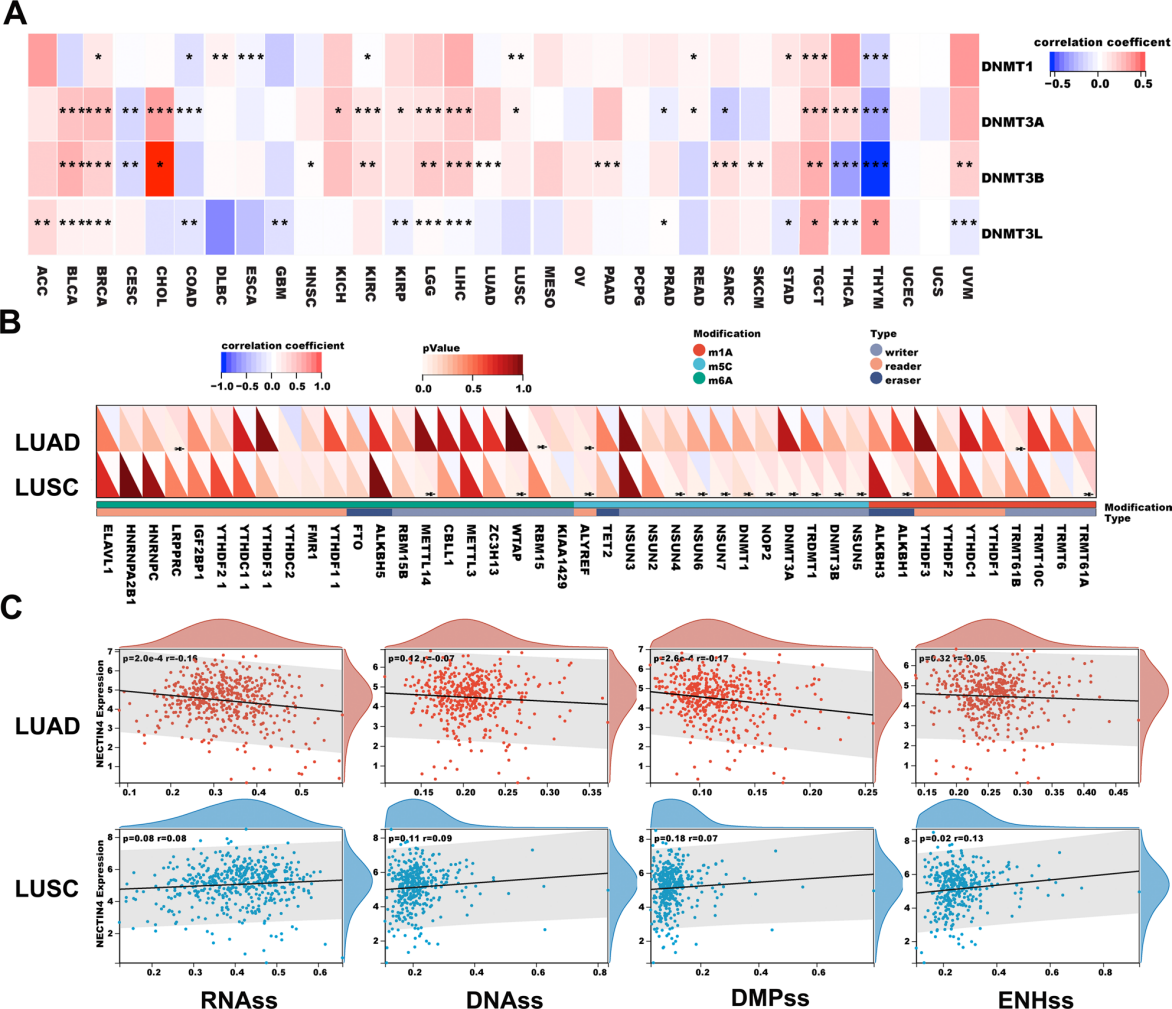
Correlation of NECTIN4 expression with DNA methylation, RNA modification, and cancer stemness. (**A**) The correlation between NECTIN4 expression and DNA methyltransferases in pan-cancer. (**B**) The correlation analysis between NECTIN4 expression and RNA modification–related genes in LUAD and LUSC. (**C**) The relationships between NECTIN4 expression and cancer stemness scores, including RNAss, DNAss, DMPss, and ENHss in LUAD and LUSC

DNA methylation is one of the most critical epigenetic modifications in cancer initiation and progression [24, 25]. As key enzymes catalyzing DNA methylation, DNA methyltransferases (DNMTs) are responsible to modulate tumor invasion, proliferation, metastasis, diagnosis, and prognosis [26–31]. In LUAD, NECTIN4 was positively correlated with DNMT1 and DNMT3B (*P* < 0.05; Fig. 4A).

In order to explore potential epigenetic regulatory pathways of NECTIN4, we examined the correlations of its expression with the genes related to different RNA modifications (Fig. 4B). Results indicated that NECTIN4 expression showed positive correlations with numerous RNA modification writers, readers, and erasers in both LUAD and LUSC. Among these, the associations with METTL3, YTHDF1, and IGF2BP3 were particularly notable, although these did not reach statistical significance.

Furthermore, we evaluated the relationship between NECTIN4 expression and cancer stemness scores in LUAD and LUSC (Fig. 4C). In LUSC, NECTIN4 expression was positively correlated with DNA stemness score (DNAss), RNA stemness score (RNAss), differentially methylated probes signature score (DMPss), enhancer methylation signature score (ENHss). In contrast, NECTIN4 expression in LUAD was negatively correlated with those scores.

### Patient characteristics

Twenty patients (6 women and 14 men; mean age 60.5 ± 8.1 years) presenting with lung lesions were enrolled. Patients diagnosed with NSCLC, SCLC, and inflammatory lesions numbered 13, 3, and 4, respectively. Table 1 presents the clinicopathological characteristics of the patients. All patients underwent paired ^18^F-FDG PET/CT and ^68^Ga-N188 PET/CT.

**Table 1:** Clinicopathological characteristics of patients

Information	Patients *n* (%)
Age (y)	
Median (range)	62 (35–72)
Sex	
Female	6 (40.0)
Male	14 (60.0)
Smoking	
No	4 (20.0)
Yes	16 (80.0)
Family history of malignancy	
No	18 (90.0)
Yes	2 (10.0)
Pathology	
Adenocarcinoma	9 (45.0)
Squamous carcinoma	4 (20.0)
Small cell lung cancer	3 (15.0)
Inflammation	4 (20.0)
Clinical Staging	
I	3 (18.8)
II	4 (25.0)
III	2 (12.5)
IV	7 (43.7)

### PET/CT uptake of lesions with different pathology

**Fig. 5:**
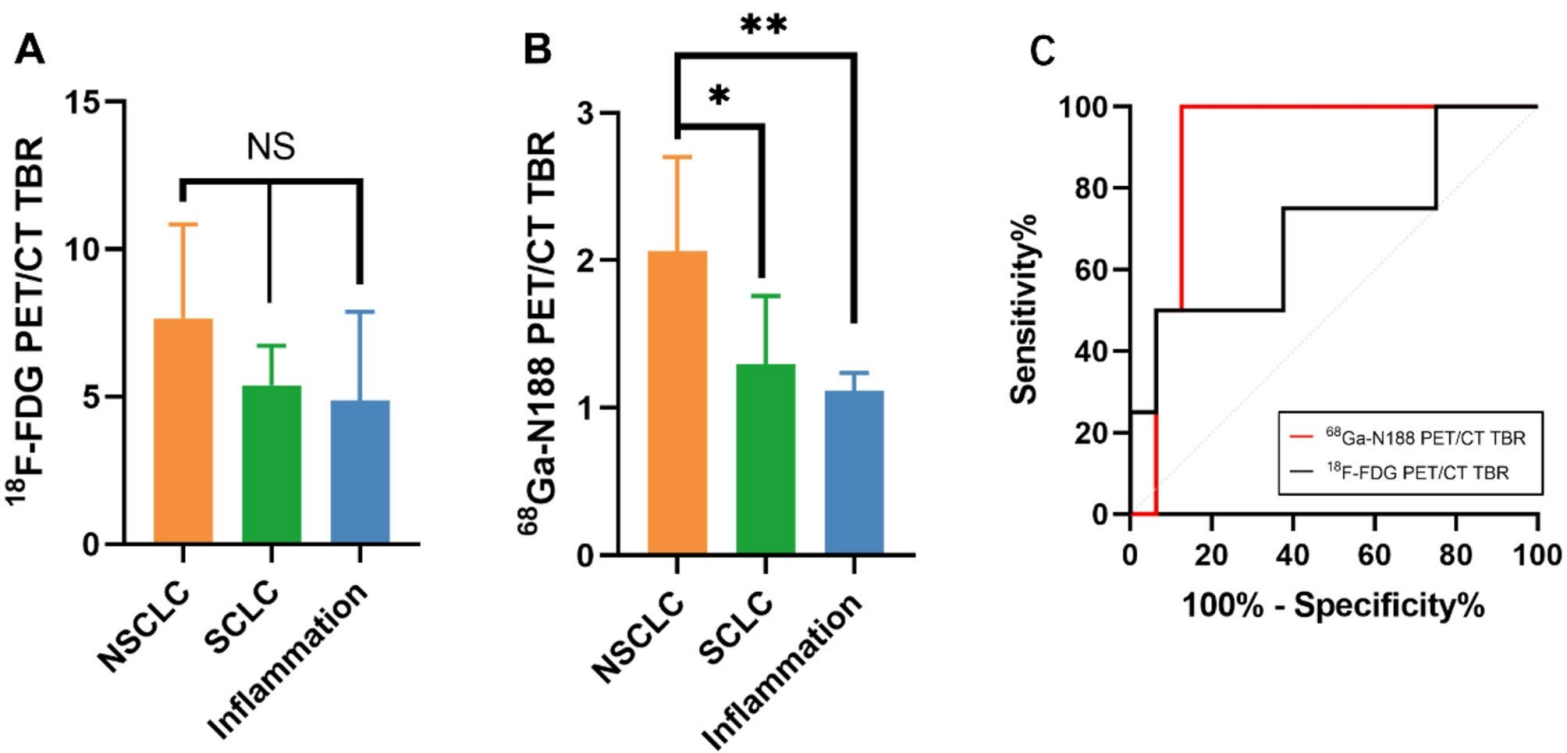
Comparison of tumor-to-blood pool ratio (TBR) and its diagnostic efficacy in ^18^F-FDG PET/CT and ^68^Ga-N188 PET/CT

**Fig. 6:**
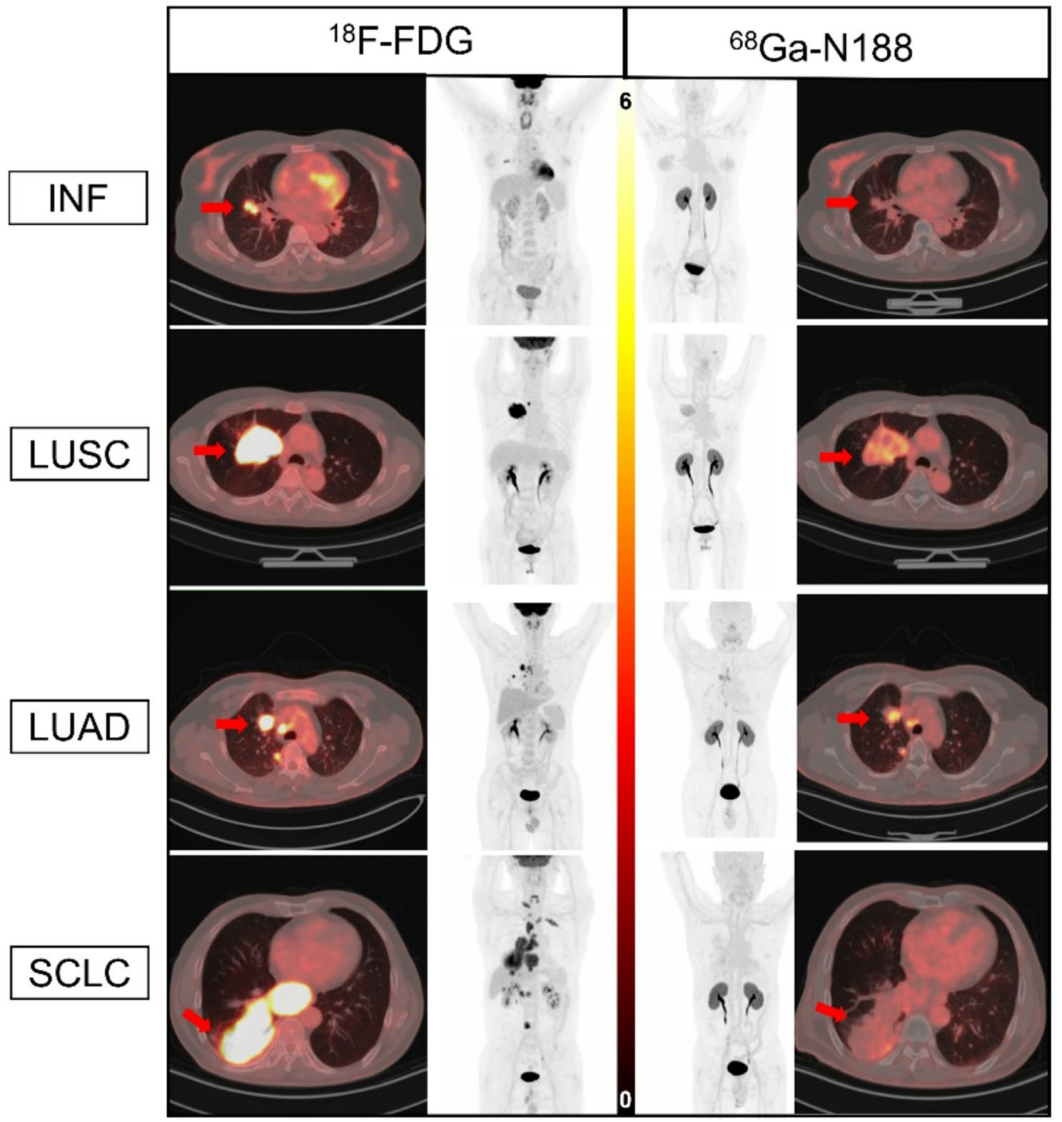
Patients with different pulmonary lesions in ^18^F-FDG PET/CT and ^68^Ga-N188 PET/CT

Using ^18^F-FDG PET/CT imaging, TBRs for NSCLC, SCLC, and inflammatory lesions were 7.7 ± 3.2, 5.4 ± 1.3, and 4.9 ± 3.0, respectively; no significant differences were observed among these groups. In ^68^Ga-N188 PET/CT imaging, tracer uptake in NSCLC was significantly higher than in SCLC (2.1 ± 0.6 vs. 1.3 ± 0.5, *P* = 0.046), and also significantly higher than in inflammatory lesions (TBR: 1.1 ± 0.1; *P* = 0.009). Figure 5A and B illustrate uptake patterns in inflammatory and malignant lung lesions, respectively, while Fig. 6 presents representative imaging examples. Figure 5C shows the ROC curves of ^18^F-FDG PET/CT and ^68^Ga-N188 PET/CT for differentiating between inflammation and cancerous lesions. The area under the curve (AUC) for ^18^F-FDG PET/CT and ^68^Ga-N188 PET/CT was 0.703 (95% CI: 0.385-1.000) (*P* = 0.219) and 0.906 (95% CI: 0.770-1.000) (*P* = 0.014), respectively. The diagnostic efficacy of ^68^Ga-N188 PET/CT was overall superior to it of ^18^F-FDG PET/CT (the sensitivity and specificity was 87.5% (95% CI: 61.7%–98.4%) and 100.0% (95% CI: 55.5%–100%) vs. 93.8% (95% CI: 69.8%–99.8%) and 50.0% (95% CI: 6.8%–93.2%), respectively.

### The detection efficacy of PET/CT for metastatic lesions in patients with lung cancer

A total of 103 lymph nodes (45 metastatic, 58 non-metastatic) were confirmed by histopathology and follow-up imaging. Using ^18^F-FDG PET/CT, 45, 15, 0, and 43 lymph nodes were classified as true positive (TP), false positive (FP), false negative (FN), and true negative (TN), respectively. In contrast, ⁶⁸Ga-N188 PET/CT identified 19 TP, 0 FP, 26 FN, and 58 TN lymph nodes. In ^18^F-FDG PET/CT, SUV_max_ values for true positive and true negative lymph nodes were 11.5 ± 3.2 and 1.9 ± 0.8, respectively (*P* < 0.001); in ^68^Ga-N188 PET/CT, the corresponding values were 4.1 ± 0.9 and 1.4 ± 0.6 (*P* < 0.001). For lymph node differential diagnosis, ^68^Ga-N188 PET/CT demonstrated higher specificity and positive predictive value (PPV) (both 100%), but lower sensitivity (42.2%). Conversely, ^18^F-FDG PET/CT showed sensitivity of 100%, specificity of 74.1%, and PPV of 75%. Detailed diagnostic efficacy metrics are summarized in Table 2.

**Table 2:** Efficacy of PET/CT in identifying metastatic lymph nodes

	Sensitivity	Specificity	Accuracy	PPV	NPV
^18^F-FDG PET/CT	100.0%	74.1%	85.4%	75.0%	100.0%
^68^Ga-N188 PET/CT	42.2%	100.0%	74.8%	100.0%	69.0%

PPV: Positive Predictive Value; NPV: Negative Predictive Value

^18^F-FDG PET/CT identified significantly more distant metastatic lesions than ^68^Ga-N188 PET/CT (51 vs. 21; mean SUV_max_ 9.2 ± 2.8 vs. 3.2 ± 0.7; *P* < 0.001) (Table 3). However, in organs characterized by high physiological ^18^F-FDG uptake—such as the brain—^68^Ga-N188 PET/CT provided superior lesion delineation compared to ^18^F-FDG PET/CT, as demonstrated by a higher TBR of 0.83 vs. 0.55.

**Table 3:** Efficacy of PET/CT in detecting metastatic lesions

	Brain	Lung	Pleura	Bone	Adrenal Gland	Others	Total (*N*)	SUV_max_
^18^F-FDG PET/CT	1	13	12	14	4	8	51	9.2 ± 2.8
^68^Ga-N188 PET/CT	1	/	12	5	3	/	21	3.2 ± 0.7

## Discussion

NECTIN4 has emerged as a biologically relevant target with potential diagnostic and therapeutic implications in cancer. Previous studies have characterized its roles in various solid tumors, demonstrating its involvement in cell proliferation, migration, and angiogenesis [32–37]. Our bioinformatic analyses revealed that NECTIN4 was overexpressed in lung cancer at both transcriptomic and proteomic levels and was associated with poor prognosis in NSCLC. Besides, NECTIN4 expression was correlated with genomic instability and epigenetic regulatory features in NSCLC. Clinically, NECTIN4 targeted ^68^Ga-N188 PET/CT showed higher specificity than ^18^F-FDG in differentiating malignant from inflammatory lung lesions, reflecting the biological relevance of NECTIN4 expression in tumor tissue.

An integrated analyses of the TCGA, GTEx, and CPTAC datasets confirmed that NECTIN4 is upregulated across multiple solid tumors, with particularly pronounced expression in NSCLC compared with SCLC. Follow-up IHC staining and western blot analyses confirmed the overexpression of NECTIN4 in lung cancer. Survival assessments utilizing data from the TCGA databases and LCE consistently indicated that patients with elevated levels of NECTIN4 experienced decreased OS. These findings support the hypothesis that NECTIN4 may serve as a molecular biomarker in NSCLC.

At the genomic level, NECTIN4 expression was positively associated with copy number amplification in NSCLC, accompanied by increased TMB, MSI, and tumor heterogeneity indices. These observations are in line with previous studies that NECTIN4 amplifications is evident in several solid tumors, most commonly in BLCA, BRCA, and LUAD, accounting for 5%–10% of all cases [38]. Moreover, in LUAD and LUSC, NECTIN4 alterations were often present in association with mutations in different cancer-related genes (TP53, TTN, RYR1, and NFE2L2), which highlights its role within complex oncogenic networks. Besides, NECTIN4 showed different trends of stemness in LUSC and LUAD. These associations may partially explain the heterogeneous expression patterns of NECTIN4 observed across lung cancer subtypes.

In addition to genomic alterations, epigenetic modifications are also instrumental in regulating the progression of cancer [39, 40]. Epigenetic analysis indicated that NECTIN4 expression may be influenced by regulatory mechanisms involving DNA methylation and RNA modifications. It is noteworthy that NECTIN4 was exhibited significant positive correlations with DNA methyltransferases DNMT1 and DNMT3B (*P* < 0.05), suggesting that DNA methylation-related regulation may influence NECTIN4 transcription. This finding was consistent with previous studies indicating that abnormal methylation mechanisms were one of the factors attributing to lung cancer progression and poor prognosis. Along with DNA methylation, NECTIN4 was also positively associated with several RNA modification regulators, such as METTL3, YTHDF1, and IGF2BP3, which implies the cross-regulatory relation between NECTIN4 and post-transcriptional m^6^A-mediated modifications [41–44] .

To investigate whether NECTIN4 expression could be used to improve the diagnostic specificity of PET/CT imaging in lung cancer, we conducted an exploratory clinical cohort study comparing the diagnostic efficacy of the NECTIN4-targeted radiotracer ^68^Ga-N188 and the standard tracer ^18^F-FDG. ^68^Ga-N188, an innovative PET imaging probe targeting NECTIN4, demonstrates high sensitivity and specificity for NECTIN4 detection in prior clinical studies. Its primary tumor identification rate is comparable to that of ^18^F-FDG PET/CT, along with improved specificity for detecting lymph node metastases across multiple cancer types [21]. In our cohort, ^68^Ga-N188 PET/CT exhibited variable uptake across patients with different pathology of lung lesions, with sensitivity and specificity values of 100% and 87.5%, respectively, in distinguishing malignant tumors from inflammatory lesions. In contrast, ^18^F-FDG PET/CT produced overlapping SUV_max_ and TBR measurements for malignant and inflammatory lesions, limiting its diagnostic accuracy. Although ^18^F-FDG PET/CT can be used with high sensitivity and specificity of 96% and 79% to distinguish between benign and malignant lung lesions, this test is prone to false-positive outcomes, especially when inflammation or granuloma exists [4, 45]. Collectively, these findings highlight ^68^Ga-N188 may provide complementary imaging modality for enhancing diagnostic precision in clinically equivocal scenarios. Although both NSCLC and SCLC lesions exhibit higher ^68^Ga-N188 uptake than inflammatory lesions, uptake in NSCLC is significantly greater than in SCLC. This finding is consistent with previous reports showing that NECTIN4 expression is predominantly upregulated in NSCLC but comparatively lower in SCLC [10]. This differential uptake suggests that ^68^Ga-N188 PET/CT may aid in molecular subtyping of lung cancer by identifying NECTIN4-enriched tumors.

Regarding lymph node metastasis, ^68^Ga-N188 PET/CT exhibits higher specificity and positive predictive value than ^18^F-FDG PET/CT, indicating its potential to reduce false-positive results. However, ^18^F-FDG PET/CT detected a greater number of metastatic lymph nodes overall, reflecting its superior sensitivity, accuracy and negative predictive value in this context. Regarding distant metastasis, 58.8% of lesions were positive on ^18^F-FDG PET/CT but negative on ^68^Ga-N188 PET/CT. However, ^68^Ga-N188 shows comparable detection performance for brain metastases. Given the well-documented limitation of ^18^F-FDG PET/CT in brain imaging—attributable to high physiological uptake in normal brain tissue—the low background signal of ^68^Ga-N188 in the brain may provide a distinct advantage for detecting intracranial lesions [46].

This study has some limitations. First, NECTIN4 expression analysis was primarily based on NSCLC data from the TCGA database, without including SCLC datasets for comparison or validation. Second, although the ^68^Ga-N188 uptake has demonstrated clinical relevance for lung cancer diagnosis, its absolute tumor uptake values were suboptimal, suggesting future optimization of probe structure to enhance imaging performance. Third, the sample size was relatively small and the absence of long-term clinical follow-up limited the evaluation of the prognostic value of ^68^Ga-N188 uptake. Future studies incorporating extended follow-up and larger patient cohorts are necessary to validate the potential of NECTIN4-targeted PET/CT as a prognostic imaging biomarker.

## Conclusions

In summary, integrative bioinformatic analyses confirm that NECTIN4 overexpression, genomic amplification, and epigenetic activation are closely associated with lung cancer progression. Correspondingly, clinical imaging studies further demonstrate that NECTIN4-targeted PET/CT using ^68^Ga-N188 outperforms conventional ^18^F-FDG PET/CT in differentiating primary lung cancer from inflammatory lesions. Collectively, these findings establish NECTIN4 as a biologically and clinically relevant molecular target in lung cancer, supporting its translational potential for improved diagnosis and targeted imaging.

## Supplementary Information

Below is the link to the electronic supplementary material.

Supplementary Material 1

Supplementary Material 2

Supplementary Material 3

Supplementary Material 4

Supplementary Material 5

Supplementary Material 6

Supplementary Material 7

Supplementary Material 8

Supplementary Material 9

Supplementary Material 10

## Data Availability

The datasets generated during and/or analyzed during the current study are available from the corresponding authors on reasonable request.

## Abbreviations

PET/CT: Positron emission tomography/computed tomography
NECTIN4: Nectin cell adhesion molecule 4
NSCLC: Non-small cell lung cancer
SCLC: Small cell lung cancers
OS: Overall survival
ADC: Antibody-drug conjugate
FDA: Food and drug administration
UC: Urothelial carcinoma
TCGA: The cancer genome atlas
GTEx: Genotype-tissue expression
CPTAC: Clinical proteomic tumor analysis consortium
scRNA-seq: Single-cell RNA-sequencing
GEO: Gene expression omnibus
HPLC: High-performance liquid chromatography
PPV: Positive predictive value
FOV: Field of view
SUVmax: Maximum standardized uptake value
SUVmean: Mean SUV
TBR: Tumor-to-blood-pool ratio
ANOVA: Analysis of variance
ROC: Receiver operating characteristic
LUSC: Lung squamous cell carcinoma
LUAD: Lung adenocarcinoma
BRCA: Breast invasive carcinoma
UCEC: Uterine corpus endometrial carcinoma
PAAD: Pancreatic adenocarcinoma
LCE: Lung cancer explorer
TP: True positive
FP: False positive
FN: False negative
TN: True negative
TMB: Tumor mutational burden
MATH: Mutant-allele tumor heterogeneity
MSI: Microsatellite instability
IHC: Immunohistochemistry
CNVs: Copy number variations
SNVs: Single nucleotide variants
DNAss: DNA stemness score
RNAss: RNA stemness score
DMPss: Differentially methylated probes signature score
ENHss: Enhancer methylation signature score
