# Supplementary material for: Bioinformatics profiling of NECTIN4 in lung cancer and comparative evaluation of NECTIN4-targeted ⁶⁸Ga-N188 and ¹⁸F-FDG PET/CT
Source: J Transl Med. 2026 Apr 27;24:759. doi: 10.1186/s12967-026-08152-8 (PMC13255257; doi:10.1186/s12967-026-08152-8)
Supplement: Supplementary file 1 — Supplementary Material 1 [file 12967_2026_8152_MOESM1_ESM.docx]

**Figure 1**. HPLC characterization of N188.
