# Supplementary figures and images for: Bioinformatics profiling of NECTIN4 in lung cancer and comparative evaluation of NECTIN4-targeted ⁶⁸Ga-N188 and ¹⁸F-FDG PET/CT
Source: J Transl Med. 2026 Apr 27;24:759. doi: 10.1186/s12967-026-08152-8 (PMC13255257; doi:10.1186/s12967-026-08152-8)

**
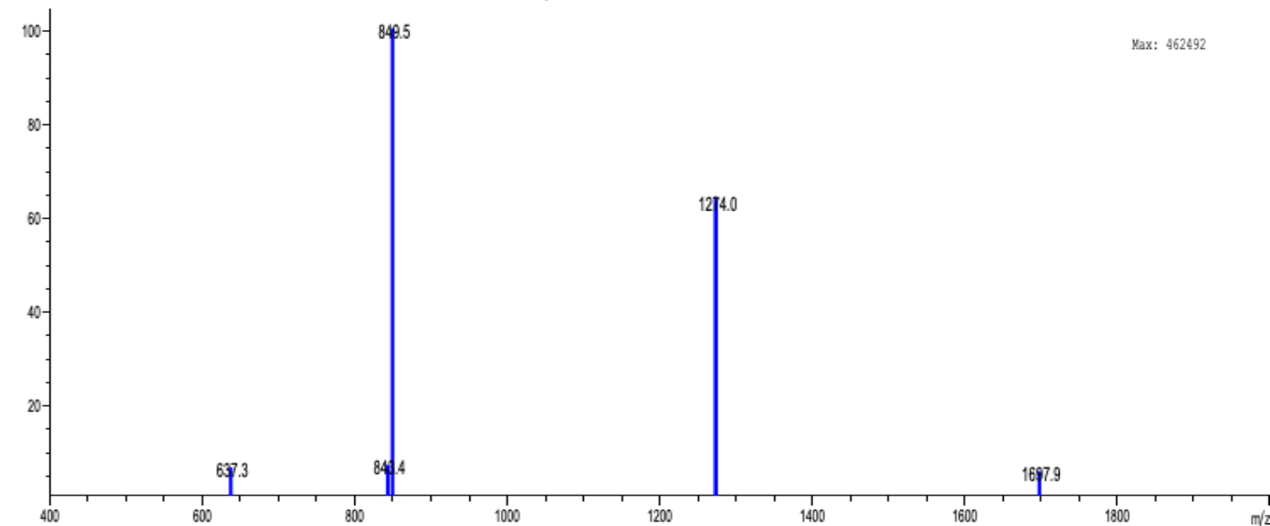
**

**Figure 2**. Mass spectrometry characterization of N188.

Supplement: Supplementary file 2 — Supplementary Material 2 [file 12967_2026_8152_MOESM2_ESM.docx]

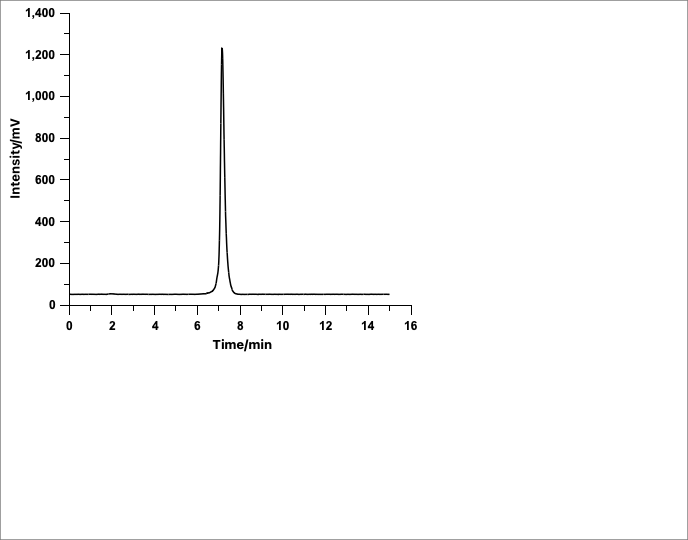


**Figure 3.** Radio**-**HPLC characterization of 68Ga-N188.

Supplement: Supplementary file 3 — Supplementary Material 3 [file 12967_2026_8152_MOESM3_ESM.docx]

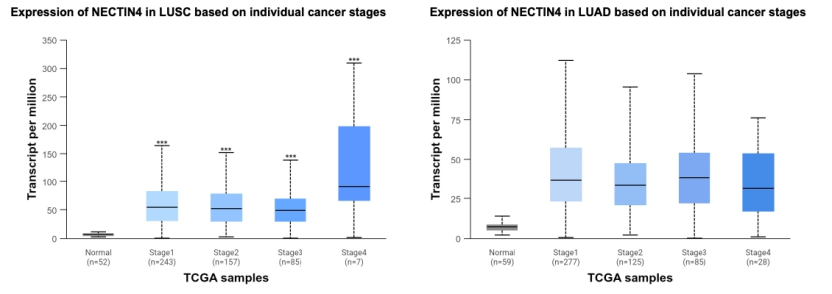


**Figure 4**. The correlation between NECTIN4 expression and NSCLC grades.

Supplement: Supplementary file 4 — Supplementary Material 4 [file 12967_2026_8152_MOESM4_ESM.docx]

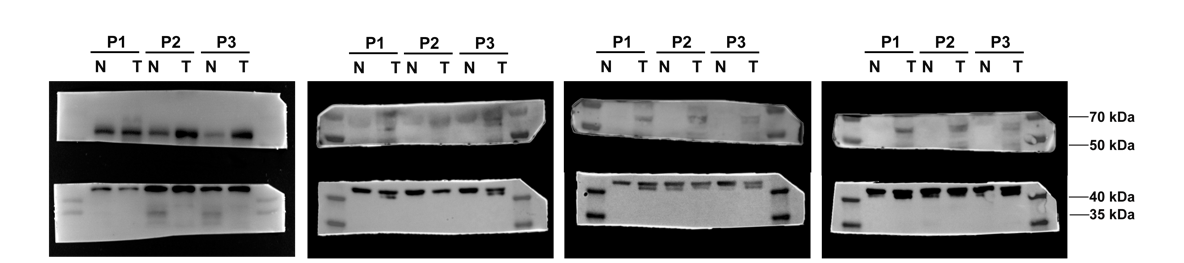


**Figure 5.** Full uncropped blots image of NECTIN4 protein expression in NSCLC.

Supplement: Supplementary file 5 — Supplementary Material 5 [file 12967_2026_8152_MOESM5_ESM.docx]
