# Supplementary material for: Bioinformatics profiling of NECTIN4 in lung cancer and comparative evaluation of NECTIN4-targeted ⁶⁸Ga-N188 and ¹⁸F-FDG PET/CT
Source: J Transl Med. 2026 Apr 27;24:759. doi: 10.1186/s12967-026-08152-8 (PMC13255257; doi:10.1186/s12967-026-08152-8)
Supplement: Supplementary file 8 — Supplementary Material 8 [file 12967_2026_8152_MOESM8_ESM.docx]

Supplementary file content

**Content**

1. **Figure 1**. HPLC characterization of DOTA-N188.
2. **Figure 2.** Mass spectrometry characterization of DOTA-N188.
3. **Figure 3.** Radio**-**HPLC characterization of ^68^Ga-N188.
4. **Figure 4.** The correlation between NECTIN4 expression and NSCLC grades.
5. **Figure 5.** Full uncropped blots image of NECTIN4 protein expression in NSCLC.
6. **Figure 6.** A meta-analysis between the expression of NECTIN4 and NSCLC from the Lung Cancer Explorer (LCE) database.
7. **Figure 7.** Correlation analyses of NECTIN4 expression with TMB, MSI, ploidy, and MATH in pan-cancer.
8. **TABLE 1. ﻿**Abbreviations
