# Supplementary material for: Bioinformatics profiling of NECTIN4 in lung cancer and comparative evaluation of NECTIN4-targeted ⁶⁸Ga-N188 and ¹⁸F-FDG PET/CT
Source: J Transl Med. 2026 Apr 27;24:759. doi: 10.1186/s12967-026-08152-8 (PMC13255257; doi:10.1186/s12967-026-08152-8)
Supplement: Supplementary file 9 — Supplementary Material 9 [file 12967_2026_8152_MOESM9_ESM.docx]

**Supplemental experimental methods:**

**Synthesis of DOTA-N188**

﻿ For synthesis of N188, 2 equivalents DOTA-tris-tert-butyl ester, activated by 2.4 equivalents HBTU, HOBt and DIPEA were added to the activated resin and reacted for 1 h at room temperature to generate intermediate **1**. Subsequently, deprotection and cleavage were performed in 5 mL mixture of trifluoroacetic acid (TFA): tri-isopropyl-silane (TIPS): water 95:2.5:2.5 (v/v/v) for 2 h. The filtrate was washed by 2 mL TFA. The solution was combined and distillated under reduced pressure to afford intermediate **2**. 50 mg intermediate **2** was dissolved in 10 mL pH = 8.0 buffer (20 mM NH_4_HCO_3_, 5 mM EDTA) with 20% acetonitrile and TATA, and reacted at 30 ^o^C for 60 min. The cyclization reaction was terminated by adding 10 equivalents cystine.

**HPLC of DOTA-N188**

**﻿** The analytical HPLC condition was eluent A (acetonitrile with 0.1% trifluoroacetic acid) and eluent B (water with 0.1% trifluoroacetic acid) at a flow rate of 1 mL/min (0-25 min, 98%-73% B; 25-25.1 min, 73%-0% B; 25.1-30 min, 0% B). Analytical HPLC were performed using a COSMOSIL Packed Column (4.6*250mm, 5μm).

**Radio-HPLC of DOTA-N188**

**﻿** The analytical HPLC condition was eluent A (acetonitrile) and eluent B (water with 0.1% trifluoroacetic acid) at a flow rate of 1 mL/min (0-1 min, 95% B; 1-10 min, 95%-5% B; 10-15 min, 5% B).
